# Undergraduate dental sleep medicine teaching at German university dental schools - a questionnaire-based survey

**DOI:** 10.1186/s12909-024-06042-5

**Published:** 2024-09-30

**Authors:** Janine Sambale, Anahita Jablonski-Momeni, Heike Maria Korbmacher-Steiner

**Affiliations:** Department of Orthodontics Clinic of Dentistry, Philipps-University Marburg, Georg-Voigt-Str. 3, 35039 Marburg, Germany

**Keywords:** Dental sleep medicine, Sleep medicine, Dental education, Dental curriculum, Undergraduate student

## Abstract

**Purpose:**

Diagnosing and treating obstructive sleep apnea (OSA) requires fundamental understanding of sleep medicine, including training and clinical experience. So far, dental sleep medicine (DSM) has not yet become a mandatory part of dental education in Germany. This questionnaire-based survey for both lecturers and students aimed to evaluate DSM education among undergraduate students.

**Methods:**

A structured questionnaire was sent to the managing directors and student councils of all 30 German university dental schools. The questionnaire contained 13 questions on teaching quantity and content, lecturers’ knowledge, and future interest in DSM. For each university dental school, only one questionnaire should be completed by the student council and the managing director. A scoring system assessed lecturers’ knowledge based on clinical experience and qualifications. Descriptive data and correlation coefficients were calculated (*P* < 0.05).

**Results:**

The responses of 24 lecturers (80%) and 28 students (93.3%) could be evaluated. DSM was reported to be included in the curriculum by 14 lecturers (58.3%) and 4 students (14.3%). Mean teaching hours per semester were 1.4 ± 1.4 h (lecturers) and 0.2 ± 0.6 h (students) accordingly. Greater knowledge of lecturers in DSM was positively correlated with the inclusion of DSM in the curriculum (*P* = 0.022) and with the number of teaching hours per semester (*P* = 0.001).

**Conclusion:**

Postgraduate education and incorporating DSM knowledge into undergraduate education (“Teach the Teacher”) seems to play a key role in fundamentally training future dentists in this field.

**Supplementary Information:**

The online version contains supplementary material available at 10.1186/s12909-024-06042-5.

## Introduction

Sleep is essential for the overall physical and mental well-being, playing an important role in optimal performance, the immune system, as well as in growth and cognitive development [1, 2]. However, sleep can often be disrupted leading to severe and even life-threatening consequences for general patient health [3–7]. Many sleep disorders manifest as oral-related conditions, which positions Dental Sleep Medicine (DSM) as a specialized field of research and clinical practice in dentistry for co-diagnosing and co-treating sleep-related breathing disorders, such as obstructive sleep apnea (OSA) [8–10]. OSA, a chronic sleep disorder characterized by repeated episodes of partial or complete obstruction of the pharyngeal airway during sleep, leads to decreased oxygen saturation and sleep disruption [11]. OSA has significant physical, mental, and social implications with many cases going undiagnosed [12, 13]. Dentists, through regular patient contact, can significantly contribute in managing this disease using myofunctional therapy, skeletal orthodontic therapeutic approaches, oral appliance therapy and upper airway surgery [4, 5, 14]. Since 2022, mandibular advancement devices (MAD) were included in statutory health insurance in Germany as a second-line therapy for patient’s intolerant to Continuous Positive Airway Pressure (CPAP). Thus, dentists are now increasingly involved in OSA treatment. However, this involvement often occurs without sufficient knowledge of the disorder, which affects not only oral health but also the overall patient’s physical and mental well-being [8].

Unfortunately, there is no or only limited DSM content in dental schools leaving the next generation of dentists inadequately prepared to handle sleep disorders [15–17]. Ivanhoe et al. [18] and Bian [19] already emphasized the necessity of education of undergraduate students. In Germany, sleep medicine has not yet become an integral, mandatory part of dental education. The German Society of Dental Sleep Medicine (Deutsche Gesellschaft Zahnärztliche Schlafmedizin: DGZS) is trying to close this gap of knowledge by offering postgraduate training in DSM for dentists interested in this field. Despite the availability of private continuing education courses, the primary issue remains that most dentists lack training in DSM [2, 20]. In university education there seems to be a pressing need to address this gap and to build a bridge between dentistry and medicine [20].

Therefore, the present questionnaire-based study aimed to evaluate sleep medicine education among undergraduate students in German dental schools. The main objectives were to:

- Evaluate the involvement of German university dental schools in DSM teaching among undergraduate students.
- Evaluate the educational teaching content from both the lecturers’ and student’s perspectives.
- Determine lecturers’ knowledge in this field and its impact on DSM teaching.

## Methods

In February 2024, a structured questionnaire was sent to the managing directors as well as to the corresponding student councils of all 30 university dental schools in Germany to evaluate academic teaching in the field of dental sleep medicine (DSM). The head of dental schools were requested to forward the questionnaire to those university faculty members who teach in the field of (dental) sleep medicine (lecturers). The student council of each dental school were asked to complete the questionnaire on behalf of the students of each semester. The questionnaire was shared as a PDF file via email as well as an online survey of the questionnaire hosted on platform “Empirio” (empirio.de). A reminder email was sent out six weeks later to call for responses to the questionnaire again, with the deadline set six weeks after the reminder email. This was followed by a personal telephone reminder over a period of two further weeks. Data was collected and anonymized prior to statistical analyses.

The questionnaires for both the lecturers (Appendix I) and the students (Appendix II) consisted of two pages and were structured in 13 main questions. Items were mainly selected according to published data of relevant topics in DSM education and to questionnaires conducted in other dental schools [15, 17, 21–23]. The authors included quantitative questions such as questions to assess teaching content, and lecturers’ and students’ future interest in this field. Additionally, the lecturers were queried about their qualifications in this field.

The questionnaire was identically for both the teaching staff and the students. The main items are listed in Table 1.

In Germany, in contrast to other countries, the number of teaching hours is recorded per semester and not per academic year. Therefore, the term “semester” was used instead of “academic year” into the questionnaire accordingly. One semester corresponds to half an academic year.

**Table 1:** Questionnaire summary: evaluated main 13 topics of (dental) sleep medicine teaching among lecturers (managing directors’ report) and students (undergraduate student councils’ report)

Included main topics of the questionnaire for the evaluation of DSM education	
**Report of DSM teaching**	Inclusion of DSM in the dental curriculum of undergraduate students yes/no
**Quantitative assessment of the inclusion of DSM in the dental curriculum**	Number of teaching hours^a^ per semester^b^ Involved departments Involved clinical staff members Involved semesters^b^ in DSM teaching
**DSM teaching content**	Fundamental basics of sleep medicine (5^c^) Sleep disorders (6^c^) Diagnostic tools and topics in adult OSA (7^c^) Diagnostic tools and topics in pediatric OSA (8^c^) Treatment of OSA (9^c^) Treatment with the MAD (10^c^) Chairside teaching in DSM
**Interest in DSM** **Lecturers’ knowledge in DSM**	Future interest in DSM Clinical experience, curricular training, and further trainings in DSM

DSM: Dental Sleep Medicine, OSA: obstructive sleep apnea, MAD: mandibular advancement device

^a^Teaching hour = one teaching hour has a duration of 45 min

^b^Semester = half an academic year

^c^ Questions 5–10 of Lecturers’ questionnaire (Appendix I)

### Statistical analysis

Statistical analyses were performed using MedCalc for Windows, version 22.021 (MedCalc Software, Ostend, Belgium). Descriptive statistics were performed for all items queried in the questionnaire. Chi-square test was used to test frequency distribution between the two categorical variables “students reported teaching hours” and “future interest in dental sleep medicine”.

A scoring system ranging from 1 to 3 was created to represent the data of lecturers’ clinical experience and additional qualifications (curriculum, training) as knowledge in (dental) sleep medicine. Taking into account the clinical experience and qualifications, a sum score was calculated:

1 = No/basic knowledge in sleep medicine (no/< 5 years clinical experience, no training or curriculum).

2 = Advanced knowledge in sleep medicine (5–10 years clinical experience or curriculum).

3 = Excellent knowledge in sleep medicine (10 years clinical experience or 5–10 years clinical experience + training 1–2 days or < 5 years clinical experience + curriculum in dental sleep medicine).

Spearman correlation coefficient (rs) was calculated to measure linear correlations between sleep medicine knowledge scores 1–3 and reported teaching hours, such as number of hours in teaching DSM per semester.

Significance level was set at α = 0.05.

## Results

Of the 30 dental schools, 24 (80%) lecturers and 28 (93.3%) student councils responded to the survey. Of the 24 responding lecturers 14 (58.3%) lecturers reported the inclusion of DSM in their dental curriculum, while four (14.3%) out of the 28 responding students reported that their dental schools included DSM in their dental curriculum.

The responses identified four departments which were involved in DSM education: The Department of Orthodontics was most frequently involved (71.4%), followed by Oral and Maxillofacial Surgery (21.4%), Prosthodontics (14.3%), and Otolaryngology (7.1%). Two teachers reported the involvement of two departments in their dental curriculum: Orthodontics and Oral and Maxillofacial Surgery, and Orthodontics and Otolaryngology.

Lecturers primarily taught DSM in the 10th (50%) and 9th (42.9%) semesters (corresponding to the final year of the undergraduate curriculum), with fewer teaching DSM in the 8th (35.7%) and 7th (21.4%) semesters. Most lecturers (57.1%) included DSM across multiple semesters, with some starting as early as the first semester. Conversely, students reported DSM being consistently included in the 7th semester (100%), followed by the 8th (75%), 9th (50%), and 10th (25%) semesters.

**Fig. 1:**
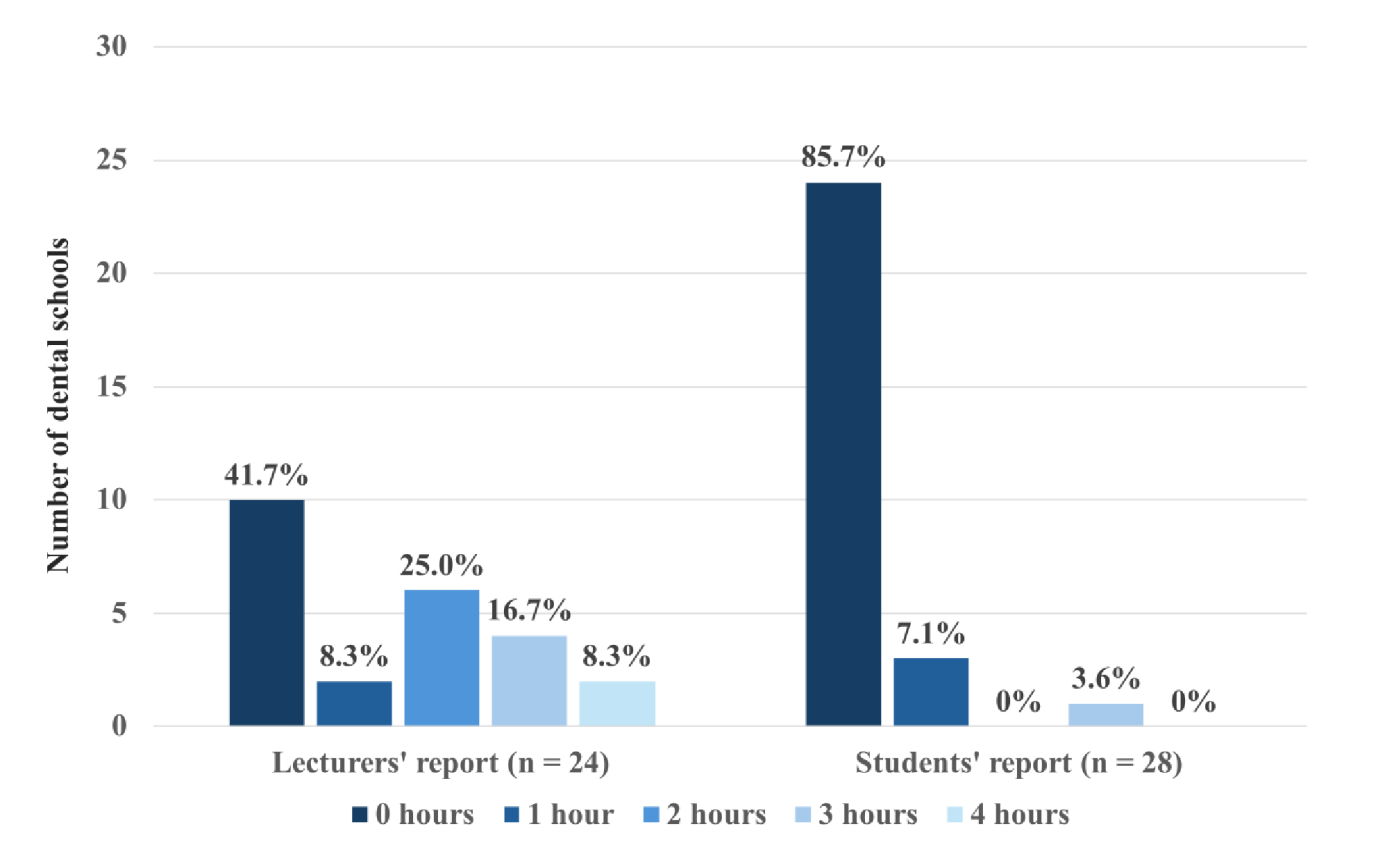
Distribution of number of teaching hours (in % in relation to the response rate) per semester at German dental schools: lecturers’ (*n* = 24) and students’ (*n* = 28) report

Regarding DSM teaching hours per semester, mean hours were 1.4 ± 1.4 h (min. 0, max. 4) as reported by the lecturers, and 0.2 ± 0.6 h (min. 0, max. 3) as reported by the students. One student mentioned that the topic was only briefly touched in connection with another subject. The distribution of the reported number of teaching hours per semester at dental schools is shown in Fig. 1.

### DSM teaching content

Lecturers reported more teaching content across all topics compared to the students. The reported teaching content differs across all topics. Among the four students who reported about DSM education at their dental schools, responses to questions 6–11 in the student questionnaire were very heterogeneous and rarely answered with „yes “. Therefore, only the percentages of lecturers‘ reports about DSM teaching content for questions 5–10 are presented in Table 2.

**Table 2:** Percentages of lecturers’ report about DSM teaching content (regarding questions 5–10)

Question No.	Teaching content	Lecturers’ report	
		Included in dental curriculum (*n*, %)	Not included in dental curriculum (*n*, %)
**(5)** ^**a**^	**Fundamental basics of sleep medicine**		
	Classification of sleep disorders	12 (50%)	12 (50%)
	Phenomenology of sleep	6 (25%)	18 (75%)
	Circadian rhythm	7 (29.2%)	17 (70.8%)
	Sleep regulation	5 (20.8%)	19 (79.2%)
	Sleep function	5 (20.8%)	19 (79.2%)
	Dream	1 (4.2%)	23 (95.8%)
	Sleep diagnostic	11 (45.8%)	13 (54.2%)
**(6)** ^**a**^	**Sleep disorders**		
	Sleep-related breathing disorders	14 (58.3%)	10 (41.7%)
	Insomnia	2 (8.3%)	22 (91.7%)
	Hypersomnia	1 (4.2%)	23 (95.8%)
	Parasomnia	1 (4.2%)	23 (95.8%)
	Circadian rhythm sleep disorders	3 (12.5%)	21 (87.5%)
	Sleep-related movement disorders	1 (4.2%)	23 (95.8%)
	Pediatric sleep disorders	5 (20.8%)	19 (79.2%)
**(7)** ^**a**^	**Diagnostic topics for OSA screening in adults**		
	Daytime sleepiness (ESS)^b^	9 (37.5%)	15 (62.5%)
	STOP BANG^c^	3 (12.5%)	21 (87.5%)
	Dental abnormalities	9 (37.5%)	15 (62.5%)
	Sleep bruxism	6 (25%)	18 (75%)
	Oral-related findings	7 (29.2%)	17 (70.8%)
	Craniofacial anomalies	13 (54.2%)	11 (45.8%)
	Comorbidities (e.g., hypertonia, diabetes)	8 (33.3%)	16 (66.7%)
**(8)** ^**a**^	**Diagnostic topics for OSA screening in children**		
	Pediatric sleep questionnaire	1 (4.2%)	23 (95.8%)
	Craniofacial anomalies	9 (37.5%)	15 (62.5%)
	Syndromes	10 (41.7%)	14 (58.3%)
	Dysfunction	6 (25%)	18 (75%)
	Oral-related findings (e.g., ankyloglossia)	4 (16.7%)	20 (83.3%)
	Behavioral abnormalities	3 (12.5%)	21 (87.5%)
**(9)** ^**a**^	**Treatment of OSA**		
	Continuous positive airway pressure (CPAP)	13 (54.2%)	11 (45.8%)
	Mandibular advancement device (MAD)	14 (58.3%)	10 (41.7%)
	Positional therapy	7 (29.2%)	17 (70.8%)
	Hypoglossal nerve stimulation	7 (29.2%)	17 (70.8%)
	Orthodontics	11 (45.8%)	13 (54.2%)
	Orthodontics and maxillofacial surgery	14 (58.3%)	10 (41.7%)
	Maxillo-mandibular advancement	11 (45.8%)	13 (54.2%)
	Combination therapy	6 (25%)	18 (75%)
	Other surgery treatment	8 (33.3%)	16 (66.7%)
	Myofunctional therapy	7 (29.2%)	17 (70.8%)
	Weight reduction	10 (41.7%)	14 (58.3%)
	Behavioral therapy	7 (29.2%)	17 (70.8%)
**(10)** ^**a**^	**Mandibular advancement device (MAD)**		
	Effects	14 (58.3%)	10 (41.7%)
	Side-effects	12 (50%)	12 (50%)
	Risk profile	6 (25%)	18 (75%)
	Indication	12 (50%)	12 (50%)
	Contraindication	10 (41.7%)	14 (58.3%)
	Splint types	6 (25%)	18 (75%)
	Bite registration	5 (20.8%)	19 (79.2%)

^a^ Questions number 5–10 of the lecturers’ questionnaire (Appendix I) regarding the teaching content

^b^ ESS = Epworth Sleepiness Scale

^c^ STOP BANG = Snoring, Tired, Observed, Pressure, Body mass index, Age, Neck size, Gender

### Future interest in dental sleep medicine

Of the 10 responding head of dental schools who did not include DSM in their dental curriculum, three dental schools (30%) did not plan to integrate DSM in the future. However, six dental schools (60%) plan to integrate DSM teaching in more than three years, while two dental schools (20%) plan to teach the topic within one year.

24 out of the 28 responding students (85.7%) reported interest in learning topics of DSM in the future, while four students at dental schools that did not teach DSM, reported no future interest in learning these topics.

There was no significant relationship between the responses of students who reported the inclusion of DSM at their dental schools and the future interest in sleep medicine knowledge, Χ^2^ (1, *n* = 28) = 0.157, *p* = 0.692.

### Lecturers’ knowledge

Of the 24 responding head of dental schools seven lecturers (29.2%) reported having no or only basic knowledge (sum score 1) in DSM. Six lecturers (25%) had advanced (sum score 2), and 11 (45.8%) had excellent knowledge (sum score 3). The number of teaching hours per semester in the field of DSM was associated with a higher score of knowledge (Table 3).

Greater knowledge in DSM was positively correlated with the inclusion of DSM in the dental curriculum (rs = 0.466, *p* = 0.022) and with the number of hours per semester teaching DSM (rs = 0.619, *p* = 0.001).

**Table 3:** Cross tabulation: association between the level of knowledge (sum score 1–3) and the number of teaching hours per semester

DSM teaching hours in *n* per semester						
Sum score	0	1	2	3	4	Prevalence in *n* (%)
**1**	5	1	1	0	0	7 (29.2%)
**2**	3	1	2	0	0	6 (25.0%)
**3**	2	0	3	4	2	11 (45.8%)
**Prevalence in n (%)**	10 (41.7%)	2 (8.3%)	6 (25.0%)	4 (16.7%)	2 (8.3%)	24 (100%)

DSM: dental sleep medicine

Sum score 1–3 = Level of teachers’ experience (1 = no/basic knowledge in DSM, 2 = advanced knowledge in DSM, 3 = excellent knowledge in DSM)

## Discussion

This questionnaire-based survey evaluated the education of (dental) sleep medicine among undergraduate students at all 30 German university dental schools. It was the first questionnaire to consider both the lecturers’ and the student councils’ perspectives on sleep medicine education. Moreover, the lecturers’ level of knowledge was taking into account.

The response rate from both lecturers’ (80%) and students’ (93.3%) were substantial and was comparable to the reported data about questionnaires distributed in other dental schools like USA (87.5%), New Zealand (85.7%), and Middle East (76%) and even higher than in France (56.2%) [15, 17, 21–23]. Therefore, the results reflect largely the overall situation of sleep medicine education at German university dental schools.

The responses of the two groups regarding the integration of dental sleep medicine into dental education differed distinctly. While 58.3% of the lecturers reported the inclusion of DSM in their dental curriculum, only 14.3% of the students reported that DSM was covered at their dental schools. One possible explanation could be that the student councils had not yet had any lessons on DSM and had also not consulted with other students in higher semesters.

In an international comparison, the integration of DSM in the dental curriculum in Germany appears to be in the lower-middle range. In France (response rate 9 out of 16 dental schools), Australia, and New Zealand (response rate 6 out of 10 dental schools), 100% of the surveyed dental schools reported that DSM was integrated into their curricula. In US, 75.5% (response rate 49 out of 56) reported DSM integration, whereas in the Middle East only 23% of the dental schools (response rate 39 out of 51) reported integration [15, 17, 21–23]. It should be noted, however, that the response rates from countries reporting 100% integration of DSM in the dental curriculum were significantly lower than in Germany. It is therefore assumed that the dental schools that did not respond have not integrated DSM into their curricula, which could result in a bias [15].

Regarding the number of teaching hours, German dental schools also seem to lack behind at first glance. The reported average number of teaching hours in the other countries was 2.5 h per academic year [24, 25]. This appears to be higher than in Germany, where the average number of teaching hours was 1.4 h. However, in our questionnaire-based survey, the teaching hours were recorded per semester, which is only half an academic year.

Lecturers who did not include DSM in their dental curriculum mostly had no or only basic knowledge of DSM. Two lecturers with excellent knowledge, who had not yet included DSM teaching, reported that they intended to integrate it within the next year. These results indicate that the training of lecturers in the field of DSM plays a key role in the teaching quantity and content of student education in this field. An insufficient level of knowledge leads to lecturers often not recognizing the impact of sleep-related breathing disorders for general patient health as well as oral health [2, 17]. Strengthening their own expertise might in turn increase their willingness to pass this knowledge on to students. Therefore, “Teach the Teacher” in the context of postgraduate training is essential to integrate a common basis and a common teaching concept with consistent content within the framework of the new licensing regulations for dentists. Regarding students’ interest in future knowledge of DSM, there was no significant correlation between students who reported the inclusion of DSM at their dental schools and their future interest in sleep medicine knowledge. However, the results showed a tendency for a lack of interest among students at dental schools where DSM was not covered in the curriculum. This could be explained by the fact that students who have no knowledge of DSM cannot evaluate the relevance of this topic for patient´s general and oral health.

There was a notable discrepancy between the reports of students and lecturers. The present study showed for the first time that the perspectives of teachers and students differed with regard to teaching DSM. Regarding DSM teaching hours per semester, students reported an average of 0.2 h which clearly differed from the lecturers’ responses. One student mentioned that the topic was only briefly addressed in connection with another subject. The low results regarding DSM teaching content of the student responses suggests that students are likely unaware when sleep medicine is taught. This discrepancy in reporting between lecturers and students further suggests that sleep medicine is not fully integrated into the dental curriculum as a separate teaching discipline in German dental schools. Therefore, the teaching content (student questions 6–11) “basics in sleep medicine”, “sleep disorders”, “diagnostic tools for OSA”, “OSA treatment”, and “Mandibular advancement device” was presented only in the lecturers’ responses in this study, whereas the four students who reported that sleep medicine was taught at their dental schools provided only little information on this topic.

In light of the findings of this questionnaire-based study, the authors have tried to identify ways in which DSM education could be enhanced, with a view to offering some practical recommendations for its improvement. The first general recommendation is the concept of “Teach the Teacher”, as mentioned above. Universities should make external training opportunities more attractive to their faculty members. In Germany, for instance, the Academy of Practice and Science (APW) offers postgraduate training programs in this field. Teachers can only effectively convey the teaching content when they possess sufficient knowledge.

The teaching content reported by the lecturers showed that they prioritized certain topics over others. While the majority of lecturers already included DSM in the curriculum, the actual content taught was partly heterogeneous. This may be due to the short teaching time, which does not allow a comprehensive coverage of sleep medicine, as it is not yet a mandatory part in the dental curriculum. All 14 lecturers who integrated sleep medicine also covered sleep-related breathing disorders and treatment with mandibular advancement devices (Table 2). However, a closer look at the teaching content reveals that not all important aspects of DSM were addressed. While for example the effects of MAD were taught by all 14 lecturers, the risk profile for MAD treatment was significantly less covered (Table 2). This is a crucial aspect of MAD treatment and it is important for future dentists to understand. Despite the inclusion of OSA treatment, particularly MAD treatment, with relatively high reported frequencies, the diagnostic topics for OSA screening in adults and children (e.g., STOP BANG, pediatric sleep questionnaires), which are highly relevant in daily clinical practice for dentists, were mostly not taught [2]. Triggs et al. [28] reported that 38.5% of orthodontists did not routinely screen their patients for OSA, attributing this to a lack of education regarding OSA. Early detection of OSA contributes to prevention oriented medical approaches that may reduce the financial burden of this increasing illness.

Basic sleep medicine content and knowledge of other sleep disorders to differentiate them from OSA, should be regarded as fundamental teaching content [26]. OSA and insomnia often coexist, interacting to amplify illness severity [27]. The co-occurrence of these two disorders, if not recognized, may complicate OSA treatment [28].

In consequence, there are some teaching contents that should be further expanded to provide a more comprehensive knowledge of sleep medicine in dental education requiring more time and an increased number of teaching hours. To achieve this, it would be desirable to integrate DSM as a separate teaching discipline including more hours of DSM teaching in the dental curriculum.

Another crucial factor influencing the quality of education in this field is interdisciplinary collaboration as DSM represents an interface between dentistry and human medicine. Practical recommendations to university faculty include the establishment of a sleep medicine board – a group of sleep physicians and dentists aimed to provide comprehensive patient care. Through this network, students could gain valuable experience by observing in sleep labs, and other medical disciplines involved such as ear nose otolaryngology, pediatric sleep medicine etc. thereby enhancing their clinical education and building new scientific working groups dealing with interdisciplinary questions.

However, it is not only the internal factors that could influence DSM education; external factors also might have a great impact. This is particularly evident in the growing awareness of the importance of sleep for the overall health: The high and increasing prevalence of OSA in the general population results in the need for an increased awareness of sleep-related oral health conditions and the necessity to gain experience in treating OSA patients with mandibular advancement devices [29].

Behrents et al. [5] presented a White Paper from the American Association of Orthodontics (AAO) that emphasizes the necessity of integrating sleep medicine, particularly the subject of OSA, into the dental curriculum. It was highlighted that it is essential for the American Dental Education Association, in collaboration with the American Dental Association and the Commission on Dental Accreditation, to establish educational standards. This ensures that OSA is taught with the appropriate endorsements and qualifications. It was also recommended that a standardized curriculum should be developed and integrated across all predoctoral and postdoctoral dental programs. Following the suggestions of this paper, it is imperative that dental and orthodontic societies in Germany also collaborate to integrate DSM into their educational frameworks. Such collaboration would ensure the development of standardized curricula and educational standards, thereby aligning with international efforts to enhance the role of sleep medicine in dental education. We would propose to include DSM education as a subfield of oral medicine in the integrated courses of the new licensing regulations [13].

### Limitations

The students’ knowledge of the teaching and content of DSM could depend on whether these student councils have already attended courses in this field or whether DSM has been integrated into the curriculum at their respective universities. The student councils from each university were asked to gather information about the knowledge of all students in each semester. However, it seems that this was somewhat challenging to organize. Factors such as communication between semester groups or individual students’ differing perceptions and memories regarding the content taught may have complicated the collection of accurate data. The authors will therefore aim to take these aspects into better consideration in future analyses to obtain an even more precise picture. One possible approach would be to record the curricular integration of DSM and the individual participation of students in corresponding courses in more detail to better understand the influence of these variables on the results and, if necessary, to control them statistically.

## Conclusion

At the 30 German dental schools the DSM content taught by lecturers varies significantly, with lecturers reporting more topics covered than students perceived. Therefore, it seems that DSM teaching has not yet been established as a separate teaching block or discipline in most dental schools in Germany. More teaching hours per semester in DSM were associated with higher knowledge scores among lecturers. Greater lecturer knowledge in DSM was positively correlated with its inclusion in the curriculum and the number of dedicated teaching hours. Thus, “Teach the Teacher” through postgraduate education and incorporating DSM knowledge into undergraduate education is crucial for training future dentists to diagnose and treat patients with sleep-related breathing disorders. Managing these disorders requires a multidisciplinary approach, offering an opportunity to bridge medicine and dentistry through comprehensive student education.

## Electronic supplementary material

Below is the link to the electronic supplementary material.

Supplementary Material 1

Supplementary Material 2

## Data Availability

All data generated or analyzed during this study are included in this article. Further inquiries can be directed to the corresponding author.
