# Supplementary material for: Undergraduate dental sleep medicine teaching at German university dental schools - a questionnaire-based survey
Source: BMC Med Educ. 2024 Sep 30;24:1074. doi: 10.1186/s12909-024-06042-5 (PMC11443930; doi:10.1186/s12909-024-06042-5)
Supplement: Supplementary file 1 — Supplementary Material 1 [file 12909_2024_6042_MOESM1_ESM.pdf]

Appendix I

Questionnaire on Student Teaching in (Dental) Sleep Medicine

Department:

Date:

1. Does your department conduct teaching sessions in the field of dental sleep medicine?
   - ☐ Yes
   - ☐ No (skip to question 12)

Questions 2-11 should only be answered if question 1 was answered with "Yes."
Multiple answers are possible.

2. Who conducts teaching sessions in the field of dental sleep medicine in your department?
   - ☐ Head of department
   - ☐ Senior physician
   - ☐ Specialized physician
   - ☐ Other staff members:____

3. In which academic year do you teach dental sleep medicine?

____

4. How many hours per semester do you teach (dental) sleep medicine?

____

5. What fundamental basics do you teach in sleep medicine?
   - ☐ Classification of sleep disorders
   - ☐ Phenomenology of sleep
   - ☐ Circadian rhythms
   - ☐ Sleep regulation
   - ☐ Sleep function
   - ☐ Dream
   - ☐ Sleep diagnostic: polygraphy, polysomnography
   - ☐ Other: ____
   - ☐ None

6. Which sleep disorders do you teach?
   - ☐ Sleep-related breathing disorders
   - ☐ Insomnia
   - ☐ Hypersomnia
   - ☐ Parasomnia
   - ☐ Circadian rhythm sleep disorders
   - ☐ Sleep-related movement disorders
   - ☐ Pediatric sleep disorders
   - ☐ Other: ____
   - ☐ None

7. What diagnostic knowledge do you teach for the screening of obstructive sleep apnea in adults?
   - ☐ Daytime sleepiness (Epworth sleepiness scale)
   - ☐ STOP BANG questionnaire
   - ☐ Dental abnormalities: ☐ Periodontitis ☐ tooth wear ☐ function (DC/TMD)
   - ☐ Sleep bruxism
   - ☐ Oral-related findings: ☐ makroglossia ☐ cheek and tongue impressions
   - ☐ Craniofacial anomalies
   - ☐ Comorbidities e.g., diabetes, hypertension, depression

- ☐ Other: ______________________________
- ☐ None

8. What diagnostic knowledge do you teach for the screening of pediatric obstructive sleep apnea?
   - ☐ Pediatric sleep questionnaire
   - ☐ Craniofacial anomalies
   - ☐ Syndromes
   - ☐ Dysfunctions
   - ☐ Oral-related findings: ☐ tonsillar hypertrophy ☐ ankyloglossia
   - ☐ Behavioral abnormalities
   - ☐ Other: ______________________________
   - ☐ None

9. Which forms of sleep therapy do you teach?
   - ☐ CPAP
   - ☐ Mandibular advancement device (MAD)
   - ☐ Positional therapy
   - ☐ Hypoglossal nerve stimulation
   - ☐ Orthodontic treatment: ☐ Maxillary expansion ☐ Functional appliance therapy
   - ☐ Combined orthodontics and orthognathic surgery
   - ☐ Maxillo-mandibular advancement
   - ☐ Combination therapy
   - ☐ Other surgery treatment (tonsillotomy/tonsillectomy, uvulopalatopharyngoplasty, nasal surgery, multi-level surgery)
   - ☐ Myofunctional therapy
   - ☐ Weight reduction
   - ☐ Behavioural therapy
   - ☐ Other: ______________________________
   - ☐ None

10. Which knowledge do you teach about mandibular advancement devices (MAD)?
    - ☐ Effects
    - ☐ Side-effects
    - ☐ Risk profile
    - ☐ Indication
    - ☐ Contraindication
    - ☐ Splint types
    - ☐ Bite registration
    - ☐ Other: ______________________________
    - ☐ None

11. Do you provide chairside instruction in dental sleep medicine?
    - ☐ Yes
    - ☐ No
    - ☐ Planned in the future, within ☐ 1 year ☐ 1-3 years ☐ > 3 years

12. Do you plan to hold courses on (dental) sleep medicine in the future?
    - ☐ Yes, within ☐ 1 year ☐ 1-3 years ☐ > 3 years
    - ☐ No

13. What qualifications do you have in this field?
    - ☐ Clinical experience ☐ < 5 years ☐ > 5 - 10 years ☐ > 10 years
    - ☐ Curricular training „Dental sleep medicine” (APW curriculum)
    - ☐ Other trainings:______________________________
    - ☐ None
    - ☐ Planned for the future, within ☐ 1 year ☐ 1-3 years ☐ > 3 years
