# Supplementary material for: Undergraduate dental sleep medicine teaching at German university dental schools - a questionnaire-based survey
Source: BMC Med Educ. 2024 Sep 30;24:1074. doi: 10.1186/s12909-024-06042-5 (PMC11443930; doi:10.1186/s12909-024-06042-5)
Supplement: Supplementary file 2 — Supplementary Material 2 [file 12909_2024_6042_MOESM2_ESM.pdf]

Appendix II

Questionnaire on Student Teaching in (Dental) Sleep Medicine

Study year:

Date:

1. Does your university conduct teaching sessions in the field of dental sleep medicine?
   - ☐ Yes
   - ☐ No (skip to question 13)

Questions 2-11 should only be answered if question 1 was answered with "Yes."
Multiple answers are possible.

2. Which department(s) teaches (dental) sleep medicine at your university?
   - ☐ Prosthodontics
   - ☐ Conservative Dentistry and Endodontics
   - ☐ Orthodontics
   - ☐ Periodontics
   - ☐ Oral surgery
   - ☐ Other: ____________________

3. Who conducts teaching sessions in the field of (dental) sleep medicine in your department?
   - ☐ Clinical director
   - ☐ Chief resident
   - ☐ Fellow
   - ☐ Resident

4. In which academic year(s) is (dental) sleep medicine taught?

____

5. How many hours per semester is (dental) sleep medicine taught?

____

6. What fundamental basics is taught in sleep medicine?
   - ☐ Classification of sleep disorders
   - ☐ Phenomenology of sleep
   - ☐ Circadian rhythms
   - ☐ Sleep regulation
   - ☐ Sleep function
   - ☐ Dream
   - ☐ Sleep diagnostic: polygraphy, polysomnography
   - ☐ Other: ____________________
   - ☐ None

7. Which sleep disorders are taught?
   - ☐ Sleep-related breathing disorders
   - ☐ Insomnia
   - ☐ Hypersomnia
   - ☐ Parasomnia
   - ☐ Circadian rhythm sleep disorders
   - ☐ Sleep-related movement disorders
   - ☐ Pediatric sleep disorders
   - ☐ Other: ____________________
   - ☐ None

8. What diagnostic knowledge is taught for the screening of obstructive sleep apnea in adults?
   - ☐ Daytime sleepiness (Epworth sleepiness scale)
   - ☐ STOP BANG questionnaire
   - ☐ Dental abnormalities: ☐ Periodontitis ☐ tooth wear ☐ function (DC/TMD)
   - ☐ Sleep bruxism
   - ☐ Oral-related findings: ☐ makroglossia ☐ cheek and tongue impressions
   - ☐ Craniofacial anomalies
   - ☐ Comorbidities e.g., diabetes, hypertension, depression
   - ☐ Other: ______________________________
   - ☐ None

9. What diagnostic knowledge is taught for the screening of pediatric obstructive sleep apnea?
   - ☐ Pediatric sleep questionnaire
   - ☐ Craniofacial anomalies
   - ☐ Syndromes
   - ☐ Dysfunctions
   - ☐ Oral-related findings: ☐ tonsillar hypertrophy ☐ ankyloglossia
   - ☐ Behavioral abnormalities
   - ☐ Other: ______________________________
   - ☐ None

10. Which forms of sleep therapy is taught?
    - ☐ CPAP
    - ☐ Mandibular advancement device (MAD)
    - ☐ Positional therapy
    - ☐ Hypoglossal nerve stimulation
    - ☐ Orthodontic treatment: ☐ Maxillary expansion ☐ Functional appliance therapy
    - ☐ Combined orthodontics and orthognathic surgery
    - ☐ Maxillo-mandibular advancement
    - ☐ Combination therapy
    - ☐ Other surgery treatment (tonsillotomy/tonsillectomy, uvulopalatopharyngoplasty, nasal surgery, multi-level surgery)
    - ☐ Myofunctional therapy
    - ☐ Weight reduction
    - ☐ Behavioural therapy
    - ☐ Other: ______________________________
    - ☐ None

11. Which knowledge is taught about mandibular advancement devices (MAD)?
    - ☐ Effects
    - ☐ Side-effects
    - ☐ Risk profile
    - ☐ Indication
    - ☐ Contraindication
    - ☐ Splint types
    - ☐ Bite registration
    - ☐ Other: ______________________________
    - ☐ None

12. Is chairside instruction provided in dental sleep medicine?
    - ☐ Yes
    - ☐ No
    - ☐ Planned in the future, within ☐ 1 year ☐ 1-3 years ☐ > 3 years

13. Would you like to learn more about (dental) sleep medicine in the future and/or do you consider the topic important?
    - ☐ Yes, within ☐ 1 year ☐ 1-3 years ☐ > 3 years
    - ☐ No
